# Hiding in plain sight: a research parasite’s perspective on new lessons in old data

**DOI:** 10.1093/gigascience/giae097

**Published:** 2024-12-04

**Authors:** Michael A Skinnider

**Affiliations:** Lewis-Sigler Institute for Integrative Genomics, Princeton University, Princeton, NJ 08540, USA; Ludwig Institute for Cancer Research, Princeton University, Princeton, NJ 08540, USA

## Abstract

High-throughput techniques that measure thousands of analytes at once have become ubiquitous features of biological research. The increasing expectation that the raw data generated by these techniques be deposited to public repositories creates rich opportunities for secondary analysis of these datasets. Such opportunities can take multiple forms. As the recipient of the 2023 Junior Research Parasite Award, I was asked to comment on the role of so-called research parasites within the ecosystem of secondary data analysis. Drawing on my own experiences, I discuss mechanisms by which reanalysis of published datasets can catalyze biological discoveries, produce resources that would be impossible to generate within a single laboratory, and drive the refinement of computational methods.

## Introduction

Over the past 2 decades, the maturation of high-throughput techniques has driven an exponential increase in the depth with which biological systems can be measured. Genomic, epigenomic, transcriptomic, proteomic, metabolomic, and lipidomic assays measure thousands of molecular phenotypes at once. Because these assays generate more measurements that can realistically be interpreted in the context of a single study, and because of expanding mandates to deposit the datasets produced by these assays in public repositories, there are more opportunities than ever to reanalyze published datasets and make new discoveries.

This is the so-called ecosystem of “research parasitism”—an ecosystem in which a (largely computational) community of “research parasites” [1] leverages datasets made publicly available by other investigators to formulate and test new hypotheses. Below, I discuss the diverse types of questions that can be addressed through secondary data analysis, drawing on my own experiences as a research parasite.

## Enabling Biological Discovery

Perhaps the most conventional form of secondary data analysis involves reanalyzing a single published dataset to address a question that was not considered by the original investigators. In the fields of mass spectrometry–based proteomics and metabolomics, which have been a major focus of my own work, opportunities of this nature are abundant. The complexity of mass spectrometric data is such that a substantial proportion of the tandem mass spectra collected in proteomic or metabolomic experiments has historically gone unidentified. Over time, however, the introduction of new computational methods and the accumulation of reference spectra have allowed increasing numbers of these previously cryptic signals to be decoded. Armed with this new knowledge, investigators can return to published datasets to identify additional analytes and correlate their abundance with phenotypes, such as disease state [2, 3].

My own work in the setting of toxicology highlights the value of returning to existing datasets long after their original collection—in this case, to reveal previously unappreciated patterns of illicit drug use [4]. In a typical year, dozens of new drugs of abuse will emerge on the illicit market. Toxicological laboratories are tasked with detecting these drugs in clinical and forensic samples to diagnose intoxications and guide public health responses. This, however, is a daunting task. Unambiguous drug identification by mass spectrometry requires synthetic standards for each drug of concern. The sheer number of new drugs that are introduced to the illicit market each year means that forensic laboratories cannot realistically acquire standards for every possible drug and must instead make difficult decisions about which standards to acquire.

My colleagues and I showed that, by reanalyzing archival mass spectrometry data from >12,000 clinical urine samples, we could uncover previously unappreciated patterns of substance use [4]. We leveraged the availability of new mass spectral data to identify a series of drugs that were not being detected by existing screens. In one particularly striking case, we discovered that the synthetic opioid fluorofentanyl had been proliferating within the community—a finding that was of significant interest to local public health officials. A subset of the identifications suggested by this secondary data analysis were validated experimentally through the acquisition of new standards, which were then used to develop new clinical assays. These efforts exemplify the potential for secondary data analysis to enable clinically relevant discoveries and guide data-driven decision-making within analytical laboratories.

## Developing Data Resources

High-throughput experiments are powerful, but they can also be expensive and labor-intensive. As a result, experiments conducted within individual laboratories typically profile a limited number of replicates—usually no more than a handful per condition. These experimental designs are often sufficient to reveal differences with large biological effect sizes but can produce both false positives and false negatives when effect sizes are smaller. A second form of research parasitism involves the meta-analysis of many small-scale experiments to reveal patterns that are reproducible across datasets. For instance, meta-analysis might reveal trends reproducible across different animal models of the same disease [5] or associated with the same physiological process in different species [6].

While meta-analysis can answer questions that are difficult to address through analysis of individual datasets, it also presents its own challenges. Experimental and computational workflows are rarely standardized across laboratories, and this variation introduces not only experimental batch effects—which have received a great deal of attention—but also computational batch effects stemming from differences in data processing.

I encountered these challenges firsthand in carrying out a meta-analysis of co-fractionation mass spectrometry (CF-MS) data [7]. CF-MS is a powerful technique for protein–protein interaction mapping, but at the time of these studies, the field had not converged on best practices for the design or analysis of CF-MS experiments. I reasoned that such best practices could be identified through a comprehensive reanalysis of all published CF-MS datasets. However, the authors of published studies had taken divergent approaches to data preprocessing, including protein identification, quantification, quality control, and normalization. To overcome the potentially confounding effects of this computational variation, I reanalyzed a total of 12,683 proteomic experiments with a standardized pipeline. This pipeline allowed us to compare different approaches to protein quantification, normalization, and quality control—all of which, we showed, could markedly impact the accuracy of downstream analysis.

Meta-analysis can also produce resources that would be impractical to assemble within a single laboratory. In the same meta-analysis of CF-MS data, and a subsequent update that more than doubled the size of this resource [8], integration of 166 human CF-MS experiments allowed us to produce one of the highest-quality maps of the human protein–protein interaction network in existence. We also inferred protein–protein interaction networks for dozens of species throughout the tree of life, in many cases for the first time. These inferences were made possible by drawing on a harmonized dataset that had required almost 2 years of uninterrupted instrument time to collect.

## Refining Computational Tools

A third form of research parasitism leverages published datasets to benchmark computational methods for the analysis of these datasets and guide the development of even better methods. My work in the setting of single-cell transcriptomics, for which the 2023 Junior Parasite Award was conferred, provides an illustrative example [9]. In this work, my colleagues and I sought to compare methods for differential expression (DE) analysis of single-cell transcriptomics data. Although similar comparisons had already been reported, these efforts had relied primarily on simulations to establish a ground truth. It seemed to us that this approach risked recapitulating the assumptions used to generate the simulated data in any resulting comparison of DE methods. We therefore sought an alternative approach.

We identified a total of 18 published experiments that collected matching bulk and single-cell RNA sequencing data from the same populations of cells exposed to the same perturbations. These datasets, we reasoned, provided a form of experimental “ground truth” that would allow for statistical methods for DE analysis of single-cell transcriptomics data to be compared on the basis of their ability to recapitulate patterns detected in the bulk datasets.

We leveraged these datasets to compare 14 of the most widely used methods for DE analysis. Surprisingly, we identified much more striking differences between statistical methods that had been apparent in simulation studies. It was apparent that all the top-performing methods shared a common property: namely, they aggregated the cells from each biological replicate before performing statistical comparisons.

Because previous benchmarks had not identified these striking differences between methods that aggregated cells from each biological replicate (“pseudobulk” DE methods) and methods that did not (“single-cell” DE methods), we again leveraged published datasets to elucidate the underlying mechanism. First, we found that single-cell DE methods were disproportionately likely to incorrectly call highly expressed genes as differentially expressed. Second, we found that randomly aggregating cells across biological samples to form “pseudo-replicates” both abolished the superior performance of the pseudobulk methods and reintroduced a bias toward highly expressed genes. Third, we showed that the common features of single-cell DE methods and DE analysis of “pseudo-replicates” arose from the tendency for statistical methods to misattribute the inherent variability between replicates to the effect of a biological perturbation. Finally, we showed that inappropriate statistical methods could produce hundreds of false discoveries even in the absence of any biological differences.

Since we first reported these findings in 2021, pseudobulk DE analysis has increasingly become the norm in the field of single-cell transcriptomics. This trend underscores the potential for research parasitism to create a “virtuous cycle”: secondary analysis of published datasets can identify optimal computational methods, and as these methods gain traction, they can in turn refine the interpretation of new datasets.

## Conclusions

Research parasites have more opportunities than ever to advance our understanding of biological systems through secondary analysis of published datasets. Secondary analyses can test new hypotheses, assemble harmonized data resources, and benchmark computational methods—and sometimes do all of the above in the same study. However, the fact that the data to be analyzed already exist does not absolve would-be parasites from the responsibility of thoughtfully negotiating the relationship between data and hypothesis [10]. Instead, parasites stand to benefit from cultivating their knowledge of the literature to identify published datasets that could address a particular question, refining their initial hypotheses based on their analysis of those datasets, and performing further experiments, either on the computing cluster or in the laboratory, to validate biological inferences and elucidate underlying mechanisms.

## Note from the Editors

The Research Parasite Awards take place at the Pacific Symposium on Biocomputing each January at the Fairmont Orchid on the Big Island of Hawaii, USA. The establishment of the award was a reaction to an editorial that presented arguments against data sharing, including that it promoted a system where “research parasites” (those who reuse datasets created by “frontline researchers”) would proliferate. As promoters of data sharing, GigaScience Press has supported the Junior Parasite Award for postdoctoral, graduate, or undergraduate trainees. Publishing Commentaries from the winners provides useful lessons for other research parasites. For more, see the Research Parasite Awards website, https://researchparasite.com/.

## Abbreviations

CF-MS: co-fractionation mass spectrometry; DE: differential expression.

## Data Availability

Not applicable.
